# Highly Efficient Bifunctional Amide Functionalized Zn and Cd Metal Organic Frameworks for One-Pot Cascade Deacetalization–Knoevenagel Reactions

**DOI:** 10.3389/fchem.2019.00699

**Published:** 2019-10-25

**Authors:** Anirban Karmakar, Anup Paul, Guilherme M. D. M. Rúbio, Mohamed M. A. Soliman, M. Fátima C. Guedes da Silva, Armando J. L. Pombeiro

**Affiliations:** Centro de Química Estrutural, Instituto Superior Técnico, Universidade de Lisboa, Lisbon, Portugal

**Keywords:** metal organic framework (MOF), crystal structure analysis, heterogeneous catalysis, cascade reaction, amide

## Abstract

A pyridine-based amide functionalized tetracarboxylic acid, 5,5′-(pyridine-2, 6-dicarbonyl)bis(azanediyl)}diisophthalic acid (H_4_L), was synthesized and its coordination chemistry toward zinc(II) and cadmium(II) ions was studied. The reactions of H_4_L with Zn(NO_3_)_2_.6H_2_O and Cd(NO_3_)_2_.4H_2_O led to its full or partial deprotonation, respectively, and the formation of the 2D coordination polymers [Zn_2_(L)(H_2_O)_4_]_n_.4n(H_2_O) (**1**) and [Cd_3_(HL)_2_(DMF)_4_]_n_.4n(DMF) (**2**) (DMF = N,N'-dimethylformamide), respectively. They were characterized by elemental analysis, FT-IR, photoluminescence, thermogravimetry, and single-crystal and powder X-ray diffraction. In **1**, the L^4−^ ligand is planar with every carboxylate anion binding a Zn(II) cation and giving rise to a 2D grid with the metals with tetrahedral environments. In **2**, the combination of bridging HL^3−^ and dimethylformamide to form trinuclear Cd(II) clusters engenders secondary building block units and generates a layer-type 2D network with the metals with octahedral and pentagonal bipyramid coordination geometries. The topological analyses of **1** and **2** reveal 2,4-connected and 3,6-connected binodal nets, respectively. On account of the presence of Lewis acid (Zn or Cd centers) as well as basic (uncoordinated pyridine and amide groups) sites, **1** and (to a much lower extent) **2** effectively catalyze the one-pot cascade deacetalization-Knoevenagel condensation reactions under quite mild conditions. They act as heterogeneous catalysts, being easy to recover and recycle without losing activity.

## Introduction

Metal organic frameworks (MOFs) are crystalline porous materials consisting of metal nodes or metal clusters connected through multitopic organic linkers, which have attracted a substantial interest recently (Karmakar et al., 2016b; Zhu et al., 2017; Abednatanzi et al., 2019; Bitzer and Kleist, 2019). The significant expansion in this area is due not only to their versatility in structures, but also to their application in different fields, such as gas storage, heterogenous catalysis, non-linear optics, biomedical application, and magnetism (Ma et al., 2009; Dhakshinamoorthy and Garcia, 2014a; Lu et al., 2014; Yuan et al., 2018).

In recent years these materials have been extensively used as heterogeneous catalysts for various organic transformations due to their extremely high surface areas, tunable pore size, easily accessible catalytic sites, high stability, and recoverability (Gascon et al., 2014; Karmakar et al., 2016a; Kang et al., 2019). Besides that, these materials can be directly implanted with catalytically active centers (acidic and basic groups) either in the ligands or metal centers, which can make them a unique material for multifunctional heterogeneous catalysis (Wen et al., 2018). In this context, our group recently developed several amide functionalized MOFs where the amide group serves as a Lewis basic center and the metal as Lewis acid, which is quite effective for catalyzing various reactions such as oxidation of alkenes and alcohols, transesterification, Knoevenagel condensation, cyanosilylation, and the Henry reaction (Karmakar et al., 2017; Karmakar and Pombeiro, 2019; Liu et al., 2019).

On the other hand, one-pot cascade reactions, where two or more reactions take place sequentially in a single pot, have attracted a significant attention recently due to their several advantages, such as the reduction of energy consumption, reaction time, waste of chemicals, and solvents (Dhakshinamoorthy and Garcia, 2014b; Huang et al., 2017; Wang et al., 2019). A particular type of cascade reaction involves the one-pot deacetalization-Knoevenagel condensation, where benzylidene malononitrile can be synthesized directly by using benzaldehyde dimethyl acetal and malononitrile. In order to achieve an effective process, it is important to use catalysts that can promote all the involved reactions (Karmakar et al., 2015; Dhakshinamoorthy et al., 2018; Zhang et al., 2019). However, the synthesis of such catalysts is not easy since different acidic and basic sites tend to neutralize each other, and the development of heterogeneous catalysts that can promote such a one-pot cascade process remains a challenge. The possibility of combining the Lewis acidic and basic properties in single MOFs provides an excellent opportunity to explore them as catalysts for such a reaction. Thus, various research groups recently tried to develop several MOFs functionalized with acidic and basic sites, which can catalyze the one-pot deacetalization-Knoevenagel reaction (Park et al., 2012; Toyao et al., 2013; Hongming et al., 2016; Liu et al., 2016; Zhang et al., 2018; Mistry et al., 2019). However, some of them required long reaction times, high catalyst loading and high reaction temperature, and the development of heterogeneous catalysts for such a reaction, which can operate under mild conditions, is still challenging and worth developing.

We have therefore synthesized an amide functionalized pro-ligand, namely 5,5′-{(pyridine-2,6-dicarbonyl)bis(azanediyl)}diisophthalic acid (H_4_L), and constructed two new 2D MOFs, [Zn_2_(L)(H_2_O)_4_]_n_.4n(H_2_O) (**1**) and [Cd_3_(HL)_2_(DMF)_4_}_n_.4n(DMF) (**2**), by using solvothermal reactions. These MOFs have been characterized by FT-IR, elemental analysis, thermogravimetric analysis, photoluminescence (Figure S1B), single-crystal, and powder X-ray diffraction analyses. Moreover, we have performed topological analysis for both the frameworks. Due to the presence of both Lewis acid (Zn or Cd centers) and basic centers (pyridyl and amide groups) these frameworks are promising candidates for bifunctional heterogeneous catalysis. Thus, we have tested their heterogeneous catalytic activity toward the one-pot cascade deacetalization–Knoevenagel condensation reaction of benzaldehyde dimethyl acetal with malononitrile under mild conditions, and a high catalytic activity was achieved. Moreover, we have also tested the recyclability and heterogeneity of these catalysts.

## Experimental

The synthetic work was performed in air and with heating. All the chemicals were obtained from commercial sources and used as received. The infrared spectra (4,000–400 cm^−1^) were recorded on a Bruker Vertex 70 instrument in KBr pellets; abbreviations: s, strong; m, medium; w, weak; bs, broad and strong; mb, medium and broad. The ^1^H NMR spectra were recorded at ambient temperature on a Bruker Avance II + 300 (UltraShield™Magnet) spectrometer operating at 300.130 MHz. The chemical shifts are reported in ppm using tetramethylsilane as the internal reference; abbreviations: s, singlet; d, doublet; t, triplet; q, quartet. Carbon, hydrogen, and nitrogen elemental analyses were carried out by the Microanalytical Service of the Instituto Superior Técnico. Thermal properties were analyzed with a Perkin-Elmer Instrument system (STA6000) at a heating rate of 5°C min^−1^ under a dinitrogen atmosphere. Powder X-ray diffraction (PXRD) was conducted in a D8 Advance Bruker AXS (Bragg Brentano geometry) theta-2-theta diffractometer with copper radiation (Cu Kα, λ = 1.5406 Å) and a secondary monochromator that was operated at 40 kV and 40 mA. Flat plate configuration was used, and the typical data collection range was between 5° and 40°. Emission spectra in solid state at room temperature were recorded on a Perkin Elmer Fluorescence Spectrometer (LS-55).

### Synthesis of 5,5′-[(Pyridine-2,6-dicarbonyl)bis(azanediyl)] Diisophthalic Acid (H_4_L)

This pro-ligand was synthesized by a two-step procedure.

In the first step, dimethyl 5-aminoisophthalate (2.09 g, 10 mmol) and NEt_3_ (1.51 g, 15 mmol) were placed in a round bottom flask and then dry CH_2_Cl_2_ (25 mL) was added, followed by the dropwise addition of 2,6-pyridinedicarboxylic acid chloride (1.01 g, 5 mmol) to the reaction mixture, and the system was stirred overnight at room temperature. Upon removal of the solvent under reduced pressure a white solid was obtained. Afterwards, water (30 mL) was added into the flask and extraction was performed with dichloromethane. The organic extracts were collected over anhydrous sodium sulfate; consequent removal of the solvent gave the methyl ester of H_4_L.

In the second step, the isolated ester (2.74 g, 5 mmol) and NaOH (0.8 g, 20 mmol) were dissolved in 25 mL of THF: water (4: 1). The reaction mixture was refluxed for 1 h at 90°C and then stirred at room temperature overnight. Afterwards, the reaction mixture was acidified (pH = 2) with dilute HCl solution. The obtained white solid product H_4_L was removed by filtration and washed with water until total removal of the acid. Anal. Calcd. for C_23_H_15_N_3_O_10_ (M = 493.38) C, 55.99; H, 3.06; N, 8.52. Found C, 62.35; H, 3.67; N, 10.42. ^1^H-NMR (DMSO-*d*_6_): 13.30 (4H, bs, -COOH), 11.41 (1H, s, -NH), 11.20 (1H, s, -NH), 8.80 (2H, s, Ar-H), 8.71 (2H, s, Ar-H), 8.20-8.41 (7H, m, Ar-H). FT-IR (KBr, cm^−1^): 3,466 (bs), 1,713 (s), 1,649 (m), 1,550 (s), 1,453 (s), 1,385 (w), 1,296 (s), 1,227 (m), 1,147 (m), 1,083 (m), 1,000 (m), 908 (w), 844 (w), 758 (s), 667 (s), 601 (m).

### Synthesis of Framework 1

H_4_L (12.3 mg, 0.025 mmol) and zinc(II) nitrate hexahydrate (14.9 mg, 0.050 mmol) were dissolved in a mixture of DMF (2 mL) and 30% ammonium hydroxide (1 mL) and then transferred and sealed in an 8 mL glass vessel and heated (solvo-thermal reactor) at 70°C for 48 h. After the reaction mixture was exposed to room temperature, small colorless crystals of **1** were obtained. Yield 63% (based on Zn). FT-IR (KBr, cm^−1^): 3,302 (bs), 1,659 (s), 1,539 (s), 1,364 (s), 1,299 (m), 1,235 (w), 1,142 (w), 1,094 (w), 994 (w), 902 (w), 764 (m), 718 (m), 653 (m), 597 (w). Anal. Calcd. for C_23_H_27_N_3_O_18_Zn_2_ (M = 764.21): C, 36.15; H, 3.56; N, 5.50. Found: C, 36.43; H, 3.45; N, 5.39.

### Synthesis of Framework 2

A solution of Cd(NO_3_)_2_.4H_2_O (15.4 mg, 0.050 mmol) and H_4_L (12.3 mg, 0.025 mmol) in 1.5 mL of DMF was prepared and then heated (hydrothermal reactor) at 75°C for 48 h in a sealed glass vessel. Block shape colorless crystals of **2** were obtained after cooling the reaction mixture to room temperature. Yield 56 % (based on Cd). FT-IR (KBr, cm^−1^): 3,262 (w), 1,702 (w), 1,647 (s), 1,612 (m), 1,542 (s), 1,370 (s), 1,321 (w), 1,280 (w), 1,109 (w), 1,021 (w), 971 (w), 908 (w), 908 (w), 777 (m), 753(m), 604 (m). Anal. Calcd. for C_70_H_80_Cd_3_N_14_O_28_ (M = 1902.69): C, 44.19; H, 4.24; N, 10.31. Found: C, 44.33; H, 4.45; N, 10.45.

### Procedure for One-Pot Cascade Deacetalization–Knoevenagel Condensation Reaction

A reaction mixture of benzaldehyde dimethyl acetal (152 μL, 1.0 mmol), malononitrile (132 mg, 2.0 mmol), and catalyst (7.6 mg of **1**, 19.0 mg of **2**, 1 mol%) was placed in a capped glass vessel, and then 1 mL DMF was added. The mixture was heated at 75°C for the desired time. Afterwards the mixture was centrifuged to remove the solid catalyst and the separated solution was extracted with CH_2_Cl_2_. The extracts were dried over anhydrous Na_2_SO_4_, whereupon evaporation of the solvent gave the crude product (2-(phenylmethylene)malononitrile), which was analyzed by ^1^H NMR after dissolving in CDCl_3_. An example of ^1^H-NMR spectra is presented in Figure S2 and the reaction yield was calculated according to a reported method (Mistry et al., 2019).

In order to perform the catalyst recycling experiments, the used catalyst (separated by centrifugation of the supernatant solution) was washed with methanol and dried in air. It was then reused for the cascade reaction as described above.

## Results and Discussion

### Synthesis and Characterization

**Scheme 1:**
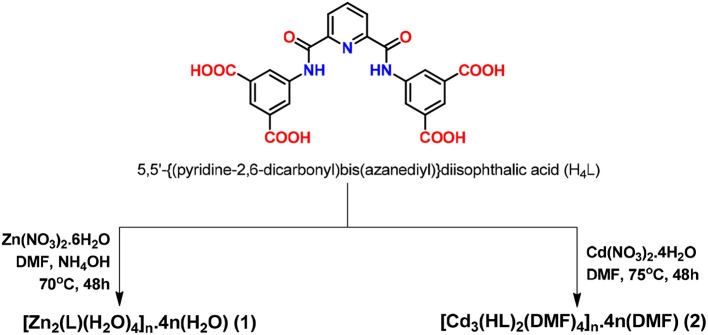
Synthesis of frameworks 1 and 2.

The solvothermal reaction of H_4_L with Zn(NO_3_)_2_.6H_2_O or Cd(NO_3_)_2_.4H_2_O in the presence or absence of NH_4_OH produced the 2D MOF [Zn_2_(L)(H_2_O)_4_]_n_.4n(H_2_O) (**1**) or [Cd_3_(HL)_2_(DMF)_4_]_n_.4n(DMF) (**2**), respectively (Scheme 1). The FT-IR spectra of **1** and **2** showed the characteristic strong bands of coordinated carboxylate groups at 1,539–1,542 cm^−1^ for the asymmetric and 1,364–1,370 cm^−1^ for the symmetric ν(CO) vibrations. The C = O stretching of amide groups appeared at 1,647–1,659 cm^−1^ and the NH of the amide groups were observed in the 3,244–3,302 cm^−1^ range. For compound **2**, the medium band at 1,702 cm^−1^ was due to ν(CO) of uncoordinated carboxylic acid group. These frameworks were characterized by microanalysis, thermogravimetry, and single crystal X-ray diffraction.

Thermogravimetric analyses were carried out under N_2_ from 30°C to *ca*. 750°C at a heating rate of 5°C min^−1^. MOFs **1** and **2** showed similar types of decomposition. **1** lost 18.9% of its weight between 30 and 330°C, most likely due to loss of non-coordinated and coordinated water molecules (calcd: 18.8%). Similarly, MOF **2** showed a continuous weight decrease of 22.8% due to loss of six DMF molecules in the temperature range of 30–269°C (calcd: 23.0%). Upon further heating, both gradually decomposed until 750°C. Thermogravimetric analyses curves for the compounds are presented in Figure S1A.

### Crystal Structure Analysis

**Figure 1:**
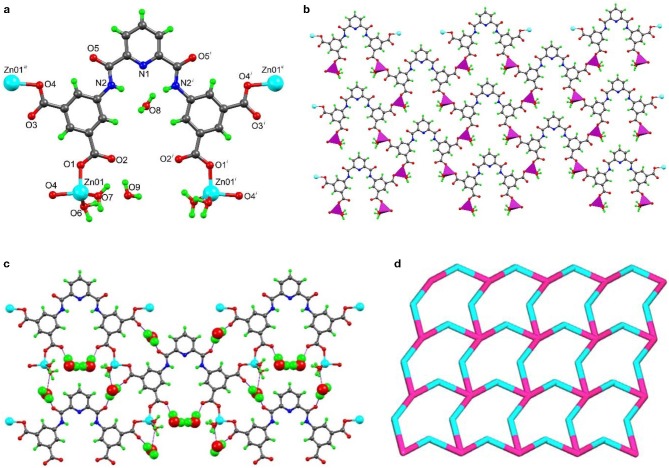
Structural representations of framework **1**: **(a)** molecular structure with partial atom labeling scheme (Symmetry codes *i* = 1–x, y, 3/2–z; *ii* = −1/2+x, 1/2+y, 1+z; and *iii* = 3/2–x, 1/2+y, 1/2–z), **(b)** two dimensional structure with polyhedral illustration, **(c)** hydrogen bonded arrangement (non-coordinated water molecules are represented as space-fill model), and **(d)** node-and-linker-type representation (the metal nodes are represented in pink and the linker L^4−^ in a cyan color).

The single crystal X-ray analysis of [Zn_2_(L)(H_2_O)_4_]_n_.4n(H_2_O) (**1**) revealed that it crystallized in the monoclinic C2/c space group (Table S1). The asymmetric unit contained one Zn^2+^ ion, half L^4−^ moiety, two coordinated and two non-coordinated water molecules (Figure 1a, Table S3). Symmetry expansion disclosed a layer-type 2D framework constructed from the deprotonated L^4−^ ligands, which bound four metal cations through one O-atom of every carboxylate group (Figure 1b). The pyridyl N-atoms remained uncoordinated. The Zn(II) center had a tetrahedral geometry (τ_4_ = 0.87) (Yang et al., 2007) where two positions were occupied by two O_carboxylate_ from two symmetry related L^4−^ ligands and the remaining ones by two water molecules. The organic ligand was planar with a maximum deviation of 0.147 Å (for O5) from the least-square plane involving all its atoms. The shortest metal–metal distance in a layer was 8.976 Å, while the shortest interlayer one was 5.806 Å. The 2D polymeric architecture of **1** has trigonal channels that are occupied by the non-coordinated water molecules (Figure 1c).

The coordinated and non-coordinated water molecules were stabilized by a number of O-H···O interactions that helped the expansion of the structure to the third dimension. Relevant hydrogen bond distances and angles are listed in Table S2. These involved the hydrogens of a non-coordinated water molecule O8 or O9 (as bifurcated donor) and the carboxylate-O or amide-O (as acceptor) via O8-H8A····O5, O8-H8B····O3, O9-H9A····O2, and O9-H9B···O9 etc. interactions (Figure 1b). The amide NH- was also involved in hydrogen bonding with non-coordinated water via N2-H1N···O8 (d_D−A_ 3.072(4) Å; < D–H····A 140°) interaction. Moreover, a number of C-H····O interactions were present in this structure, which helped its expansion to the third dimension.

**Figure 2:**
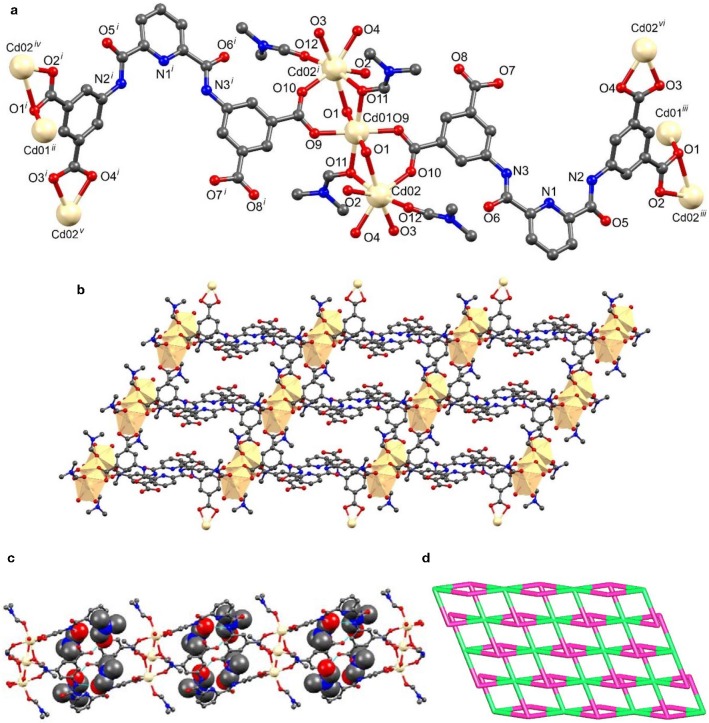
Structural representations of framework **2**: **(a)** molecular structure with partial atom labeling scheme (symmetry codes *i* = –x, 1–y, –z; *ii* = x−1, y, z−1; *iii* = 1+x, y, 1+z; *iv* = x, y, 1+z; −1–x, 1–y, −1–z; *v* = −x, 1–y, −1–z and *vi* = x, y, 1+z), **(b)** two dimensional structure with polyhedral illustration, and **(c)** 2D packing diagram viewed down the crystallographic c axis with hydrogen-bonded DMF molecules (non-coordinated DMF molecules are represented in space fill model), **(d)** node-and-linker-type depiction (the metal nodes are represented in green and the linker HL^3−^ in a pink color).

The asymmetric unit of [Cd_3_(HL)_2_(DMF)_4_]_n_.4n(DMF) (**2**) contained two Cd(II) ions, one of them (Cd1) sat on an inversion center, one triply deprotonated ligand (HL^3−^), two coordinated and two non-coordinated DMF molecules (Figure 2a, Table S3). Symmetry expansion revealed that **2** is also a layer, the HL^3−^ ligand coordinated simultaneously to five different metal ions through the carboxylate groups which act as chelate bidentate (κ-*OO'*), bridging (μ), and bridging-chelate (μ, 1κ-*OO'*,2κ-*O'*) donors. The cadmium cations present different geometries, one is octahedral (Cd1) and the other is pentagonal bipyramidal (Cd2). In both cases the O_caboxylate_ atoms from two (in the case of Cd2) or three (for Cd1) symmetry related HL^3−^ ligands occupied the equatorial positions, and the axial sites are engaged with DMF molecules; these, in turn, can act as monodentate and monodentate-bridging ligands.

In this framework, the HL^3−^ organic ligand was highly twisted. The least-square plane of the carboxyl containing isophthalate ring made a dihedral angle of 12.46° with that of the pyridyl ring and an angle of 49.40° with the other isophthalate moiety. The two crystallographically independent Cd-ions were arranged in trinuclear clusters, forming [Cd_3_(COO)_6_] assemblies as secondary building blocks (Figure 2b). The Cd1····Cd2 distance within the tri-nuclear clusters was of 3.5146 Å. Association of six HL^3−^ ligands and tri-nuclear Cd(II) cluster results in the formation of a 2D framework (Figure 2b).

The non-coordinated DMF molecules were hydrogen bonded with both amine and carboxyl groups of HL^3−^ ligand through N-H····O and O-H····O interactions (Figure 2c).

We have also performed the topological analysis of frameworks **1** and **2** by reducing their multidimensional structures to simple node-and-linker nets using TOPOS 4.0 (Blatov, 2012). Topological analysis of framework **1** reveals that it has a 2,4-connected binodal net with point symbol {8^4^.12^2^}{8}_2_ (Figure 1d). In the case of framework **2**, we have considered the trinuclear Cd(II) unit as a single node and connected with six different HL^3−^ ligands, of which each ligand connected with three Cd(II) clusters. Thus, framework **2** has the 3,6-connected binodal net with point symbol of {4^3^}_2_{4^6^.6^6^.8^3^} (Figure 2d).

### Catalytic Activity Studies Toward One-Pot deacetalization–Knoevenagel Reactions

**Scheme 2:**
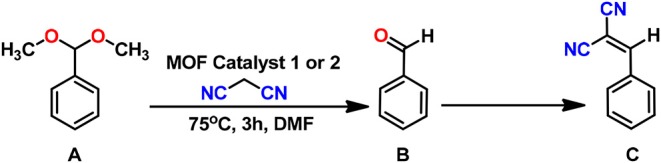
One-pot deacetalization-Knoevenagel condensation reactions.

Our synthesized frameworks **1** and **2** possessed Lewis acid metal sites (Zn^2+^ and Cd^2+^), which catalyze the deprotection of benzaldehyde dimethyl acetal to give benzaldehyde. Moreover, they also contained free pyridyl-N and amide groups (basic sites) that can promote the Knoevenagel condensation reaction of benzaldehyde and malononitrile. Thus, these frameworks were a kind of potential bifunctional acid-base catalysts. In addition, their high thermal stability and insolubility in common solvents made them suitable to act as heterogenous catalysts. To prove it, we have explored their catalytic activities as heterogeneous catalysts for the one-pot cascade deacetalization-Knoevenagel condensation reactions (Scheme 2).

In a typical reaction, a mixture of benzaldehyde dimethyl acetal (0.152 g, 1.0 mmol), malononitrile (0.132 g, 2.0 mmol) and catalyst (7.6 mg for **1** and 19.0 mg for **2**; 1.0 mol %) in 0.5 mL of DMF was confined in a capped glass vessel and was stirred at 75°C for the desired time. The reaction mixture was then centrifuged to remove the solid catalyst and the organic products were extracted with dichloromethane. Evaporation of the solvent from the extract produced crude products, which were assessed by ^1^H NMR (Figure S3).

We have optimized the reaction conditions by changing the solvents, temperature (21–75°C), catalyst loading (0.5–2 mol %) and reaction time (up to 3 h), and the obtained results are presented in Table 1.

**Table 1:** Optimization of the parameters of the one-pot deacetalization–knoevenagel condensation reactions between benzaldehyde dimethyl acetal and malononitrile with **1** and **2** as the catalysts^a^.

**Entry**	**Catalyst**	**Time (h)**	**Amount of catalyst (mol%)**	***T* (^**°**^C)**	**Solvent**	**Yield of unreacted A (%)*^**b**^***	**Yield of B (%)*^**b**^***	**Yield of C (%)*^**b**^***
1	**1**	3	1	75	DMF	1	0	99
2	**2**	3	1	75	DMF	12	31	57
3	**1**	0.25	1	75	DMF	72	13	15
4	**1**	0.5	1	75	DMF	48	27	25
5	**1**	0.75	1	75	DMF	40	28	32
6	**1**	1	1	75	DMF	30	21	49
7	**1**	1.5	1	75	DMF	19	14	67
8	**1**	2	1	75	DMF	8	7	85
9	**1**	2.5	1	75	DMF	4	3	93
10	**2**	0.5	1	75	DMF	45	40	15
11	**2**	1	1	75	DMF	20	60	20
12	**2**	1.5	1	75	DMF	16	50	34
13	**2**	2	1	75	DMF	14	41	45
14	**2**	3	1	75	DMF	12	31	57
**Different solvents**								
15	**1**	3	1	75	THF	45	55	0
16	**1**	3	1	75	MeOH	54	28	18
17	**1**	3	1	75	CH_3_CN	34	34	32
18	**1**	3	1	75	EtOH	41	43	16
19	**1**	3	1	75	DMSO	19	21	58
20	**1**	3	1	75	Solvent free	13	22	65
21	**2**	3	1	75	THF	76	24	0
22	**2**	3	1	75	MeOH	92	0	8
23	**2**	3	1	75	CH_3_CN	57	31	12
24	**2**	3	1	75	EtOH	83	11	6
25	**2**	3	1	75	DMSO	21	47	32
26	**2**	3	1	75	Solvent free	18	48	34
**Different temperatures**								
27	**1**	3	1	25	DMF	60	40	0
28	**1**	3	1	50	DMF	43	57	0
29	**1**	3	1	100	DMF	3	7	90
30	**2**	3	1	25	DMF	83	17	0
31	**2**	3	1	50	DMF	55	45	0
32	**2**	3	1	100	DMF	7	42	51
**Catalyst amount**								
33	**1**	3	0.5	75	DMF	47	12	41
34	**1**	3	2	75	DMF	2	0	98
35	**2**	3	0.5	75	DMF	45	14	41
36	**2**	3	2	75	DMF	8	35	57
37	Blank	3	–	75	DMF	82	18	0
38	Zn(NO_3_)_2_.6H_2_O	3	1	75	DMF	39	56	5
39	Cd(NO_3_)_2_.6H_2_O	3	1	75	DMF	45	53	2
40	H_4_L	3	1	75	DMF	80	20	0

a *Typical reaction conditions: 1 mol% of catalyst **1** or **2**, DMF (0.5 mL), benzaldehyde dimethyl acetal (152 mg, 1.0 mmol) and malononitrile (132 mg, 2.0 mmol), 75°C*.

b *Calculated by ^1^H NMR analysis*.

***A**, benzaldehyde dimethyl acetal; **B**, benzaldehyde; **C**, benzylidene malononitrile*.

**Figure 3:**
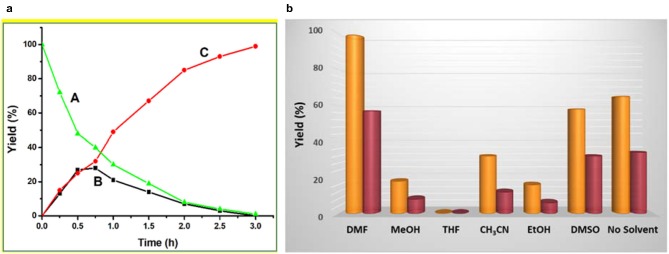
**(a)** Plot of yield vs. time for the one-pot cascade deacetalization-Knoevenagel condensation reactions catalyzed by framework **1** [red line: yield of 2-benzylidenemalononitrile (C); black line: yield of intermediate benzaldehyde (B); green line: yield of unreacted benzaldehyde dimethyl acetal (A). **(b)** Yield of 2-benzylidenemalononitrile in different solvents catalyzed by frameworks **1** (yellow pillar) and **2** (crimson pillar).

Performing the cascade reaction in different solvents, such as DMF, MeOH, THF, CH_3_CN, EtOH, DMSO, and without any solvent (Figure 3b), indicated that DMF (99% yield for **1** and 57% yield for **2**) was the best one (entries 1–2, Table 1). By using tetrahydrofuran (THF), benzaldehyde dimethyl acetal converted into benzaldehyde but no benzylidene malononitrile was formed (entries 15 and 21, Table 1). The use of ethanol (EtOH) lead to the lowest yield (*ca*. 6–16%) of benzylidene malononitrile, followed by MeOH (*ca*. 8–18% yield), CH_3_CN (*ca*. 12–32%) and DMSO (*ca*. 32–58%) (entries 16–19 and 22–25, Table 1). Under solvent-free conditions, 34–64% of benzylidene malononitrile yield was obtained (entries 20 and 26, Table 1). The yields observed with the mentioned solvents were much lower than those obtained with DMF (99%), which thus turned out to be the best solvent. Moreover, these studies revealed that framework **1** had a significantly higher catalytic activity than **2** under similar experimental conditions on account of the higher Lewis acidity of Zn(II) in **1** compared to Cd(II) in framework **2**.

Optimization of the reaction temperature is also important as this parameter significantly influences the final yield. Thus, in order to examine the effect of temperature, the reaction was carried out at different temperatures. No benzylidene malononitrile was formed at a temperature at least up to ca. 50°C (entries 27–28 and 30–31, for **1** and **2**, respectively, Table 1). However, increasing the reaction temperature to 75°C resulted in 99% (for **1**) and 57% (for **2**) conversion of benzaldehyde dimethyl acetal into benzylidene malononitrile (entries 1 and 2, respectively, Table 1). Further increase in the temperature to 100°C had a negative effect on the yield (Table 1, entries 29 and 32).

In order to find out the best catalyst loading, the catalyst amount was varied in the range of 0.5–2 mol % using the abovementioned experimental conditions. For 0.5 mol% of catalyst **1** at 75°C, the yield of 41% of 2-benzylidenemalononitrile was obtained (entry 33, Table 1), whereas 1 mol % of catalyst **1** resulted in a remarkable yield increase to 99% (entry 1, Table 1). However, a further increase in the catalyst amount to 2 mol % did not result in a further yield enhancement (98%, entry 34, Table 1), indicating that 1 mol% of catalyst **1** is the best fit for the reaction. In the case of **2**, an increase in the catalyst loading from 0.5 mol to 1 mol% produced an yield increase from 41 to 57% (entries 35 and 2, Table 1), which also did not enhance upon further increasing the catalyst load to 2% (entry 36, Table 1).

In order to find the most adequate time, the reaction was monitored until 3 h at regular intervals. The kinetic plots of 2-benzylidenemalononitrile (**C**) and benzaldehyde (**B**) yields [and of the relative amount of benzaldehyde dimethyl acetal (**A**)] vs. time are shown in Figure 3a for **1** and in Figure S2 for **2**. The final product (**C**) yield increased up to 3 h, beyond which only a rather slow yield increase would occur. Moreover, the yield of the benzaldehyde intermediate (**B**) increased with time until reaching a maximum at *ca*. 0.8–1.0 h. The relative amount of the substrate [(**A**), benzaldehyde dimethyl acetal] continuously decreased with time. These behaviors are typical for an **A**→**B**→**C** reaction sequence, as was expected.

Blank tests were also performed by eliminating the use of any metal catalyst, and no 2-benzylidenemalononitrile was formed under the same experimental conditions (entry 37, Table 1). A rather low yield of 2-benzylidenemalononitrile (5 or 2%) was observed using the metal salt Zn(NO_3_)_2_.6H_2_O or Cd(NO_3_)_2_.6H_2_O (entries 38 and 39, respectively, Table 1). On the other hand, using H_4_L instead of the metal catalyst did not result in any conversion (Table 1, entry 40).

A comparison of the catalytic activity of our catalyst **1** with other reported MOFs toward one-pot deacetalization-Knoevenagel condensation reactions is shown in Table 2, indicating that our catalyst is possibly the most active one. For example, using [Cd_3_(SIPA)_2_(ABPY)_3_(DMF)_2_]_n_. (BPDB). (DMF)_2_ as a catalyst resulted into an overall yield of 95% (lower than ours) after 5 h reaction time at 80°C (entry 2, Table 2) (Mistry et al., 2019). Moreover, the use of a 3D Sm(III) MOF and of the Al-MOF [MIL-101(Al)-NH_2_] lead to a yield of 76 and 94%, respectively (entries 6 and 7, Table 2) (Toyao et al., 2013; Zhang et al., 2018). In the case of [Zn_4_(TBCB).v (H_2_O)_6_]_n_.5n(DMAc), a yield of 99% was achieved at 90°C after 4 h (entry 3, Table 2), a higher reaction time and temperature than ours (Hongming et al., 2016). Various Cu(II) frameworks such as Cu-PCN-124 and Cu-HNUST-8 lead to the same yield (99%) of catalyst **1** but required a longer time, i.e., 12 and 48 h, respectively (entries 4 and 5, Table 2) (Park et al., 2012; Zheng et al., 2018).

**Table 2:** A comparison of catalytic activity of various MOFs in the one-pot deacetalization-Knoevenagel condensation reactions.

**Entry**	**Catalyst**	**Solvent/Temp/Time/catalyst amount**	**Yield (%)**	**References**
1	**1**	DMF/75°C/3 h/1 mol%	99	This work
2	[Cd_3_(SIPA)_2_(ABPY)_3_(DMF)_2_]_n_. (BPDB). (DMF)_2_	DMF/80°C/5 h/0.5 mol%	95	Mistry et al., 2019
3	[Zn_4_(TBCB). (H_2_O)_6_]_n_.5n(DMAc)	1,4-Dioxane/90°C/4 h/6 mol%	99	Hongming et al., 2016
4	Cu-PCN-124	DMSO/50°C/12 h/0.5 mol%	99	Park et al., 2012
5	Cu-HNUST-8	DMSO/50°C/48 h/0.5 mol%	99	Zheng et al., 2018
6	[(CH_3_)_2_NH_2_]_2_[Sm_6_(μ^3^-OH)_8_(BDC-NH_2_)_6_(H_2_O)_6_]·x(solvent)	DMSO-d_6_/50°C/24 h/ 2 mol%	76	Zhang et al., 2018
7	MIL-101(Al)-NH_2_	1,4-Dioxane/90°C/3 h/3 mol%	94	Toyao et al., 2013

*SIPA, 5-sulfoisopthalate; ABPY, 4,4′-azopyridine; BPDB, 1,4-bis(4-pyridyl)-2,3-diaza-1,3-butadiene; TBCB, 2,2′,6,6′-tetrakis[3,5-bis-3,5-benzenedicarboxylate]benzidine; DMAc, N,N-dimethylacetamide; BDC-NH_2_, 2-amino terephthalic acid; Cu-PCN-124, 3D Cu(II)- 5,50-[(pyridine-3,5-dicarbonyl)bis (azanediyl)]diisophthalate framework; Cu-HNUST-8, 3D Cu(II)- 4′,4^‴^-{(pyridine-3,5-dicarbonyl)bis(azanediyl))bis(([1,1′-biphenyl]-3,5 dicarboxylate} framework; MIL-101(Al)-NH_2_, 3D Al-2-amino terephthalate MOF*.

**Figure 4:**
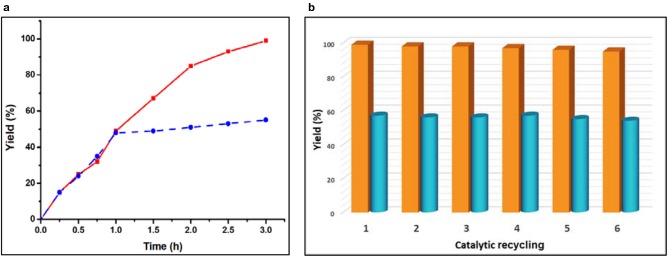
**(a)** Plot of yield vs. time for the one-pot cascade deacetalization-Knoevenagel condensation reactions catalyzed by framework **2** [red line: yield of 2-benzylidenemalononitrile (**C**); dotted blue line: yield of 2-benzylidenemalononitrile (**C**) after removing the catalysts after 1 h of reaction time]. **(b)** Effect of the catalyst recycling on the yield of 2-benzylidenemalononitrile from the one-pot cascade deacetalization-Knoevenagel reactions catalyzed by **1** (yellow pillar) and **2** (cyan pillar).

A desired relevant property of a heterogeneous catalyst concerns its reusability. In order to check this behavior, our catalysts were recovered after the catalytic reaction, washed and dried again for reuse. They could be reused successively at least six times without significant loss to their catalytic activity (Figure 4b). In order to check the heterogeneity of each of our catalysts, we removed it by centrifugation after 1 h and kept the catalyst-free reaction solution under the same conditions, stirred for an additional 2 h. As shown in Figure 4a (blue dotted line), after removal of the solid catalyst **1**, the product yield did not increase significantly. We also observed a similar phenomenon in the case of catalyst **2** (Figure S2). This establishes the heterogeneous nature of our catalysts (Lempers and Sheldon, 1998). Moreover, we also determined the amount of Zn(II) and Cd(II) ions in the solution after removing the catalysts from the reaction mixture and found only a negligible value (0.015–0.018% of the amount used in the reaction), which indicated that no leaching occurred during the catalytic process. The structural integrity of the catalysts after the catalytic process was also checked by FT-IR and powder X-ray diffraction analyses was performed before and after their use (Figures S4, S5). The identities between the FT-IR spectra and between the powder XRD diffractograms supported that the structure of both catalysts remained intact after the catalytic reaction.

## Conclusions

We have successfully designed, synthesized and fully characterized the two new 2D frameworks [Zn_2_(L) (H_2_O)_4_]_n_.4n(H_2_O) (**1**) and [Cd_3_(HL)_2_ (DMF)_4_]_n_.4n(DMF) (**2**). They contain both Lewis acid (Zn or Cd center) and Lewis basic (amide and free pyridyl groups) sites, which makes them suitable for bifunctional catalysis. Indeed, those MOFs act as heterogeneous catalysts for the one-pot cascade deacetalization–Knoevenagel reactions of benzaldehyde dimethyl acetal and malononitrile to produce 2-benzylidene malononitrile in high yields (99% within 3 h of reaction time, in the case of **1**). Framework **1** is the most active one, possibly due to the higher Lewis acidity of zinc relative to cadmium. These catalysts are stable under the catalytic conditions and can be recycled at least up to six times without a significant loss of activity. This work reveals that an amide functionalized Zn(II) MOF can act as an efficient heterogeneous bifunctional catalyst for the above cascade reactions under considerably mild conditions, aspects that are of significance in green and sustainable chemistry. On the other hand, our Cd(II) MOF (**2**) did not exhibit a significant catalytic activity for such a type of bifunctional catalysis. Furthermore, due to the toxicity associated with cadmium, its compounds should be used cautiously, even when behaving as heterogenous catalysts. The study also contributes to a better understanding of the usefulness of MOFs as platforms for the design of new acid–base bifunctional heterogenous catalysts.

## Data Availability Statement

All datasets generated for this study are included in the manuscript/Supplementary Files.

## Author Contributions

AK: overall planning, synthesis and characterization of MOFs and ligand, catalytic studies, manuscript writing. APa: catalytic studies and Photoluminescence studies. GR: synthesis of ligand and MOFs. MS: catalytic studies. MG: single crystal X-ray diffraction analysis. APo: manuscript reading and correcting.

### Conflict of Interest

The authors declare that the research was conducted in the absence of any commercial or financial relationships that could be construed as a potential conflict of interest.
