# Supplementary material for: Highly Efficient Bifunctional Amide Functionalized Zn and Cd Metal Organic Frameworks for One-Pot Cascade Deacetalization–Knoevenagel Reactions
Source: Front Chem. 2019 Oct 25;7:699. doi: 10.3389/fchem.2019.00699 (PMC6824099; doi:10.3389/fchem.2019.00699)
Supplement: Supplementary file 1 [file Data_Sheet_1.docx]

**Supporting Information**

**Highly Efficient Bifunctional Amide functionalized Zn and Cd Metal Organic Frameworks for One-pot Cascade Deacetalization−Knoevenagel Reactions**

**Anirban Karmakar*, Anup Paul, Guilherme M. D. M. Rúbio, Mohamed M.A. Soliman, M. Fátima C. Guedes da Silva and Armando J. L. Pombeiro***

Centro de Química Estrutural, Instituto Superior Técnico, Universidade de Lisboa, Av. Rovisco Pais, 1049–001, Lisbon, Portugal. E-mail: [*anirbanchem@gmail.com*](mailto:anirbanchem@gmail.com); [*pombeiro@tecnico.ulisboa.pt*](mailto:pombeiro@tecnico.ulisboa.pt)*.*

**EXPERIMENTAL**

**Instruments**

The synthetic work was performed in air and with heating. All the chemicals were obtained from commercial sources and used as received. The infrared spectra (4000–400 cm^-1^) were recorded on a Bruker Vertex 70 instrument in KBr pellets; abbreviations: s = strong, m = medium, w = weak, bs = broad and strong, mb = medium and broad. The ^1^H NMR spectra were recorded at ambient temperature on a Bruker Avance II + 300 (UltraShield^TM^Magnet) spectrometer operating at 300.130 MHz. The chemical shifts are reported in ppm using tetramethylsilane as the internal reference; abbreviations: s = singlet, d = doublet, t = triplet, q = quartet. Carbon, hydrogen and nitrogen elemental analyses were carried out by the Microanalytical Service of the Instituto Superior Técnico. Thermal properties were analyzed with a Perkin-Elmer Instrument system (STA6000) at a heating rate of 5˚C min^-1^ under a dinitrogen atmosphere. Powder X-ray diffraction (PXRD) was conducted in a D8 Advance Bruker AXS (Bragg Brentano geometry) theta-2-theta diffractometer, with copper radiation (Cu Kα, λ = 1.5406 Å) and a secondary monochromator, operated at 40 kV and 40 mA. Flat plate configuration was used and the typical data collection range was between 5˚ and 40˚. Emission spectra in solid state at room temperature were recorded on a Perkin Elmer Fluorescence Spectrometer (LS-55).

**Crystal structure determinations**

X-ray quality single crystals of the compounds (**1** and **2**) were immersed in cryo-oil, mounted in a Nylon loop and measured at room temperature. Intensity data were collected using a Bruker APEX-II PHOTON 100 diffractometer with graphite monochromated Mo-Kα (λ 0.71069) radiation. Data were collected using phi and omega scans of 0.5° per frame and a full sphere of data was obtained. Cell parameters were retrieved using Bruker SMART (Bruker, 2012) software and refined using Bruker SAINT (Bruker, 2012) on all the observed reflections. Absorption corrections were applied using SADABS (Sheldrick et al., 1996). Structures were solved by direct methods by using the SHELXS-2014 package (Sheldrick et al., 2015) and refined with SHELXL-2014/6 (Sheldrick et al., 2015). Calculations were performed using the WinGX System-Version 2014.1 (Farrugia et al, 2012). The hydrogen atoms attached to carbon and nitrogen atoms were inserted at geometrically calculated positions and included in the refinement using the riding-model approximation; U_iso_(H) were defined as 1.2U_eq_ of the parent atoms for phenyl and 1.5U_eq_ of the parent atoms for the methyl groups and nitrogen atoms. The hydrogen atoms of coordinated water molecules and carboxylic acid were located from the final difference Fourier map and the isotropic thermal parameters were set at 1.5 times the average thermal parameters of the belonging oxygen atoms. Least square refinements with anisotropic thermal motion parameters for all the non-hydrogen atoms and isotropic ones for the remaining atoms were employed. Crystallographic data are summarized in Table S1 (Supplementary Information file) and selected bond distances and angles are presented in Table S2. CCDC 1946368-1946369 contain the supplementary crystallographic data for this paper. These data can be obtained free of charge from The Cambridge Crystallographic Data Centre *via* [www.ccdc.cam.ac.uk/data_request/cif](http://www.ccdc.cam.ac.uk/data_request/cif).


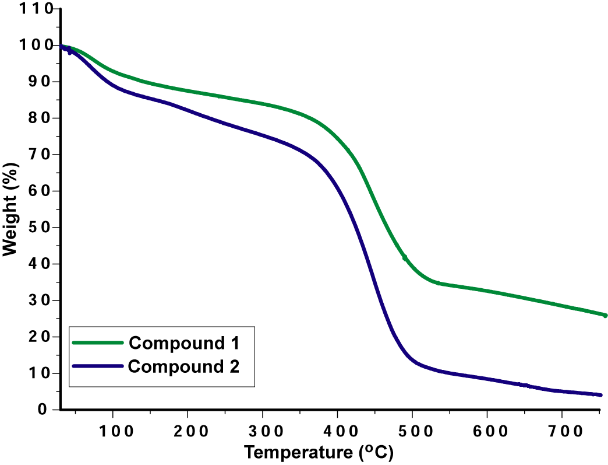

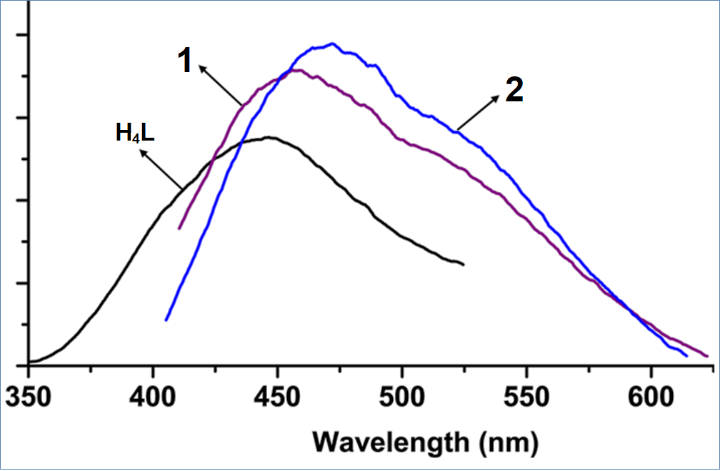


A B

Figure S1 A) Thermogravimetric curves of frameworks **1** and **2**. B) The solid-state emission spectra of the **H_4_L**, **1** and **2** at room temperature.

**Photoluminescence studies**

Metal organic frameworks having d^10^ metal ions can display interesting photoluminescence properties with application as photoactive materials and chemical sensors (Ding et al., 2012; Bauer et al., 2007). Thus, the photo-luminescence properties **1** and **2** were inspected in the solid state at room temperature, along with H_4_L for comparison (Figure S1B, supporting information). The emission peak observed at 444 nm for the free ligand (λ_ex_ = 300 nm) is attributed to the π-π* of the intra-ligand transition (Xu et al., 2014). On the other hand, **1** and **2** exhibit photo-luminescence with an emission band at 462 or 471 nm respectively, with a red shift of *ca.* 30 nm upon excitation at λ_ex_ = 360 nm. The emission band observed for **1** and **2** may be ascribed to intra-ligand charge transfer transition and the slight increase in emission intensity in both **1** and **2** in comparison to the free ligand can be due to metal−ligand coordination, which may be accounted for by the increase of the rigidity of the ligand (Xu et al., 2014; Wang et al., 2005).


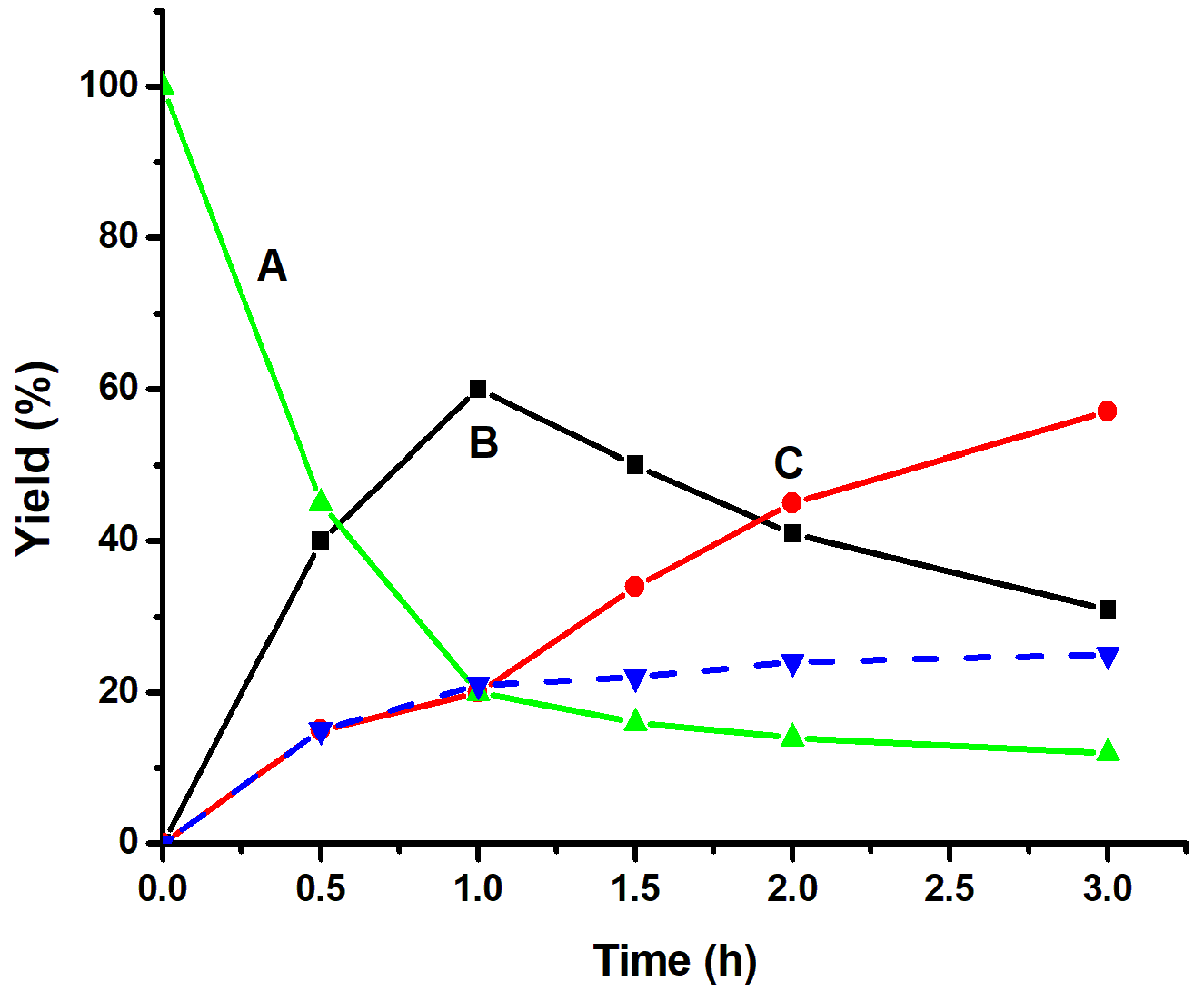


Figure S2: Plot of yield vs. time for the one-pot tandem deacetalization-Knoevenagel condensation reactions catalysed by framework **2** [red line: yield of 2-benzylidenemalononitrile (**C**); black line: yield of intermediate benzaldehyde (**B**); green line: yield of unreacted benzaldehyde dimethyl acetal (**A**); dotted blue line: yield of 2-benzylidenemalononitrile (**C**) after removing the catalysts after 1h of reaction time].


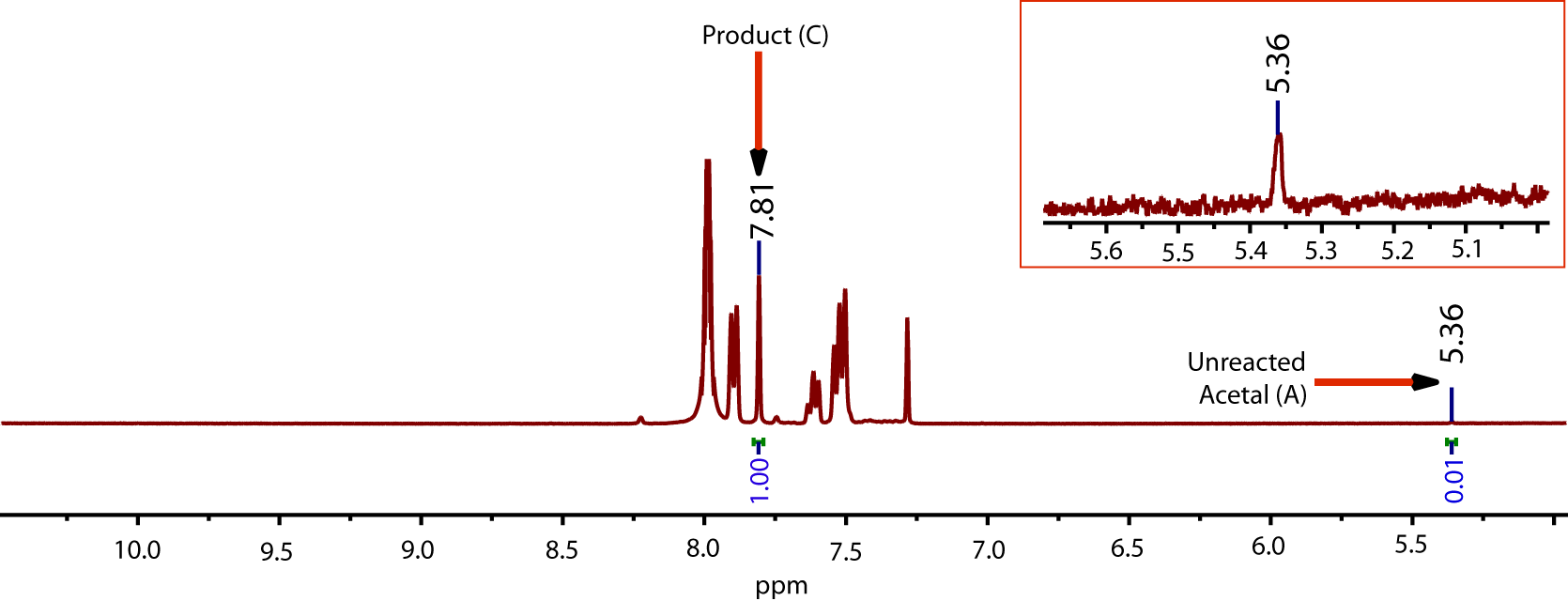


A


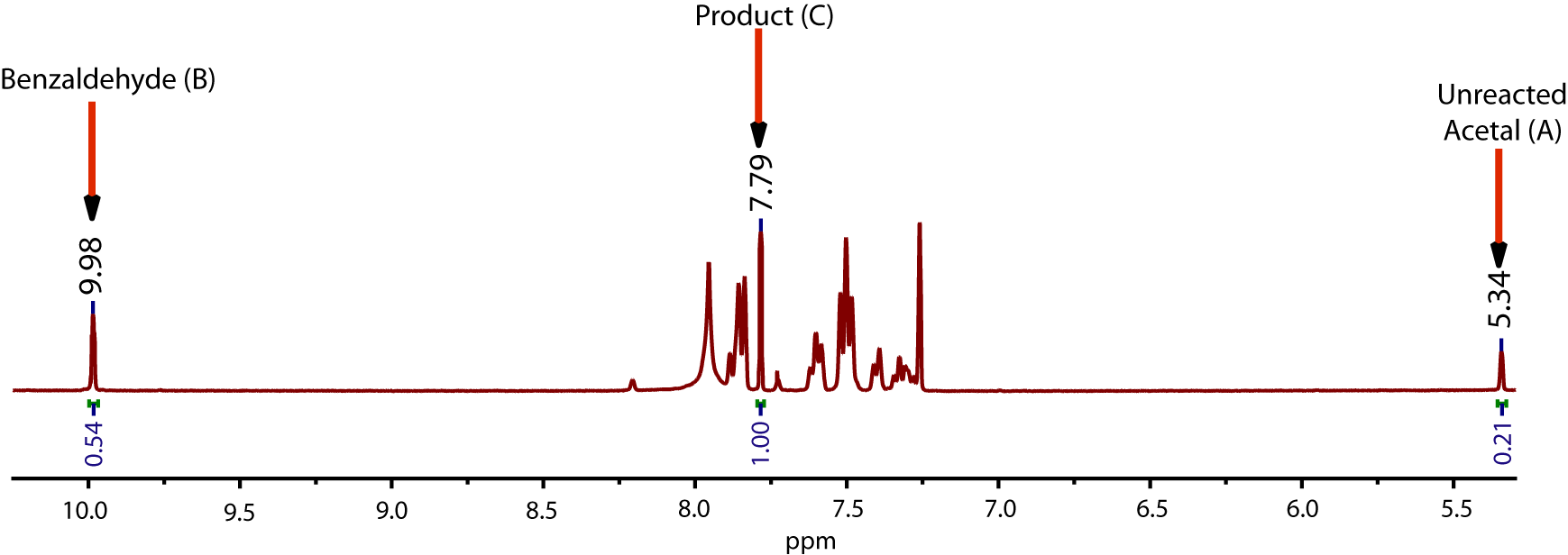


B

Figure S3 Example of integration in the ^1^H-NMR spectrum for the determination of the product yield in the one-pot tandem deacetalization−Knoevenagel condensation reactions catalysed by **1** (A) [Table 1, entry 1] and **2** (B) [Table 1, entry 2].


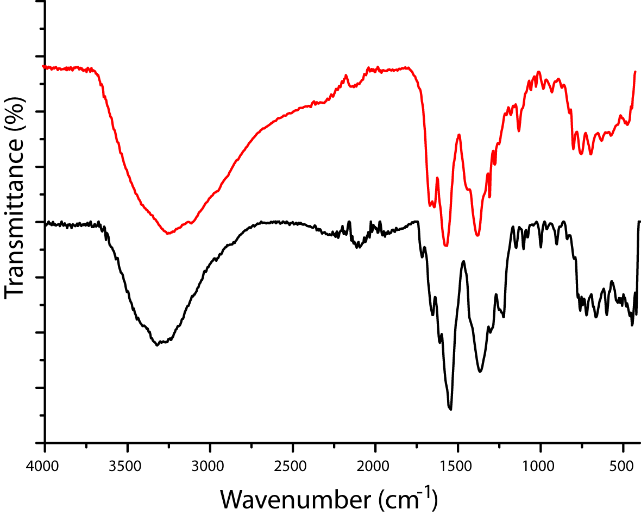

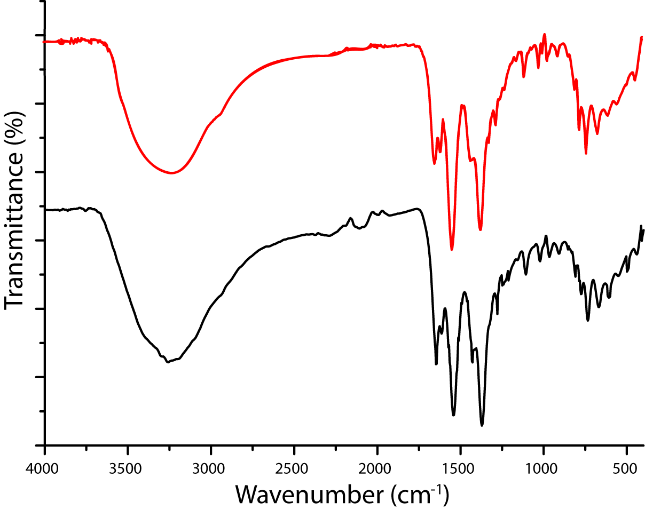


A B

Figure S4 (A) FT-IR spectra of **1** before (in black) and after the cascade reactions (in red). (B) FT-IR spectra of **2** before (in black) and after the cascade reactions (in red).


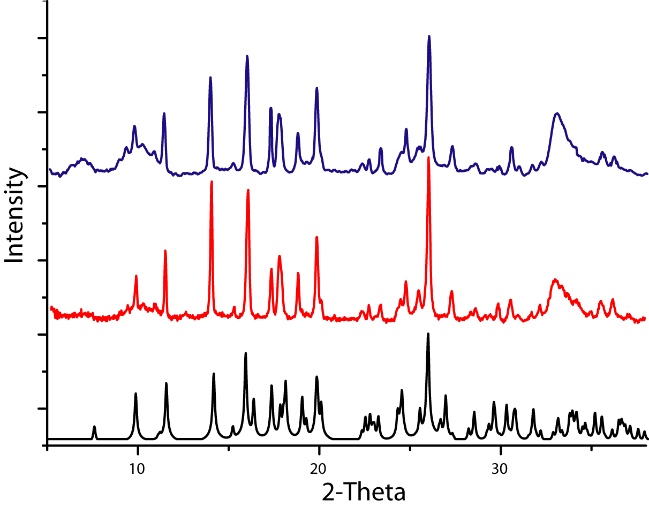

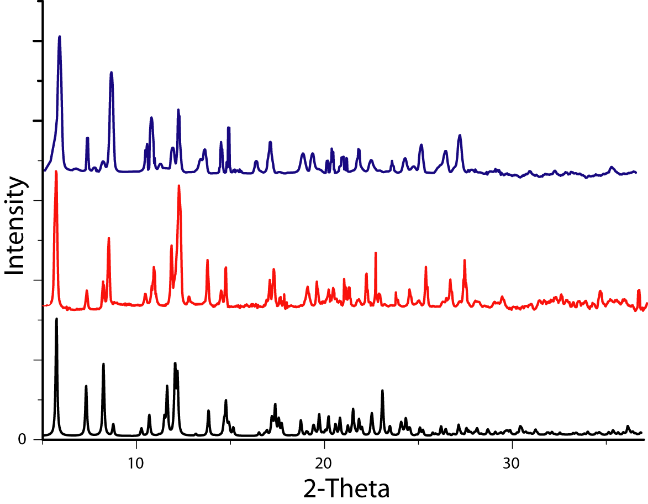


A B

Figure S5 Powder XRD diffractograms of **1** (A) and **2** (B) simulated (in black), as synthesized (in red) and after the cascade reactions (in blue).

| **Table S1: Crystal data and structure refinement details for compounds 1 and 2** | | |
| --- | --- | --- |
| Identification name | **1** | **2** |
| Formulae | C_23_H_27_N_3_O_18_Zn_2_ | C_70_H_80_Cd_3_N_14_O_28_ |
| Mol. wt. | 764.21 | 1902.68 |
| Crystal system | Monoclinic | Triclinic |
| Space group | C2/c | P-1 |
| Temperature /K | 296 | 296 |
| Wavelength /Å | 0.71073 | 0.71073 |
| *a /Å* | 17.8935(7) | 10.1045(4) |
| *b /Å* | 15.2885(7) | 12.3922(5) |
| *c /Å* | 10.8146(5) | 15.8063(7) |
| α/° | 90 | 102.6970(10) |
| β/° | 90.742(2) | 92.042(3) |
| γ/° | 90 | 91.6280(10) |
| V/ Å^3^ | 2958.2(2) | 1928.26(14) |
| Z | 4 | 1 |
| Density/Mgm^-3^ | 1.716 | 1.639 |
| Abs. Coeff. /mm^-1^ | 1.710 | 0.912 |
| F(000) | 1560 | 966 |
| Refl. collected | 19403 | 34795 |
| Refl. unique | 2734 | 7103 |
| Max. 2θ/° | 25.451 | 25.444 |
| Ranges (h, k, l) | -21<= h <=21  -18<= k <=18  -13<= l <= 13 | -12 <= h <=12  -14 <= k <=14  -19 <= l <= 19 |
| Complete to 2θ (%) | 99.8 | 99.7 |
| Refl. with I > 2σ(I) | 2437 | 6447 |
| Data/Restraints/Parameters | 2734/9/245 | 7103/18/546 |
| Goof (*F^2^*) | 1.055 | 1.090 |
| R1 [I > 2s(I)] | 0.0335 | 0.0249 |
| wR2 [I > 2s(I)] | 0.0918 | 0.0596 |
| R1 [all data] | 0.0386 | 0.0294 |
| wR2 [all data] | 0.0963 | 0.0618 |

| **Table S2: Hydrogen bond geometry (Å, °) in compounds 1-2** | | | | | | |
| --- | --- | --- | --- | --- | --- | --- |
| Compound | D-H∙∙∙A | D∙∙∙H (Å) | H∙∙∙A (Å) | D∙∙∙A (Å) | <D−H∙∙∙A(°) | Symmetry codes |
| **1** | N2-H1N∙∙∙N1 | 0.73 | 2.34 | 2.716(3) | 114 | - |
|  | N2-H1N∙∙∙O8 | 0.73 | 2.48 | 3.072(4) | 140 | x, -y+1, z+1/2 |
|  | O8-H8A∙∙∙O5 | 0.88 | 1.89 | 2.774(3) | 176 | - |
|  | O8-H8B∙∙∙O3 | 0.88 | 2.02 | 2.889(4) | 168 | -x+3/2, y+1/2, -z+1/2 |
|  | O9-H9B∙∙∙O9 | 0.91 | 1.87 | 2.770(8) | 166 | -x+1, y, -z+3/2 |
|  | O9-H9A∙∙∙O2 | 0.92 | 1.90 | 2.786(4) | 161 | x, y+1, z |
|  | O7-H7A∙∙∙O3 | 0.93 | 2.20 | 3.094(4) | 162 | x, -y, z+1/2 |
|  | O6-H6A∙∙∙O8 | 0.92 | 2.26 | 2.996(4) | 136 | x, y-1, z |
|  | O6-H6B∙∙∙O2 | 0.94 | 2.32 | 3.048(4) | 134 | -x+1, -y, -z+1 |
|  | O7-H7B∙∙∙O4 | 0.93 | 2.56 | 3.429(4) | 157 | -x+3/2, -y+1/2, -z+1 |
|  | C6-H6∙∙∙O5 | 0.93 | 2.21 | 2.821(3) | 122 | - |
|  |  |  |  |  |  |  |
| **2** | O7-H7∙∙∙O14 | 0.82 | 1.82 | 2.637(3) | 174 | x-1, y, z |
|  | N2-H2N∙∙∙O13 | 0.86 | 2.23 | 3.065(3) | 165 | - |
|  | N3-H3N∙∙∙N1 | 0.86 | 2.29 | 2.709(3) | 110 | - |
|  | N3-H3N∙∙∙O13 | 0.86 | 2.27 | 2.986(3) | 141 | - |
|  | C6-H6∙∙∙O5 | 0.93 | 2.29 | 2.844(3) | 117 | - |
|  | C23-H23∙∙∙O6 | 0.93 | 2.23 | 2.842(3) | 123 | - |
|  | C24-H24∙∙∙O9 | 0.93 | 2.39 | 3.075(3) | 130 | - |
|  | C11-H11∙∙∙O5 | 0.93 | 2.49 | 3.395(3) | 166 | -x+2, -y, -z+1 |
|  | C26-H26C∙∙∙O3 | 0.96 | 2.42 | 3.206(4) | 139 | x-1, y, z-1 |
|  | C30-H30∙∙∙O8 | 0.93 | 2.56 | 3.249(4) | 132 | x+1, y, z |
|  | C29-H29C∙∙∙O4 | 0.96 | 2.53 | 3.256(4) | 133 | - |
|  | C28-H28C∙∙∙O7 | 0.96 | 2.58 | 3.315(4) | 134 | - |
|  | C25-H25A∙∙∙O14 | 0.96 | 2.60 | 3.398(5) | 141 | -x+1, -y+1, -z+1 |
|  | C25-H25B∙∙∙O6 | 0.96 | 2.50 | 3.225(5) | 132 | x-1, y, z |
|  | C35A-H35A∙∙∙O1 | 0.96 | 2.53 | 3.338(6) | 141 | -x+1, -y, -z+1 |
|  | C34A-H34A∙∙∙O3 | 0.96 | 2.59 | 3.166(8) | 119 | -x+1, -y, -z+1 |
|  | C34A-H34C∙∙∙O6 | 0.96 | 2.16 | 3.111(9) | 168 | - |
|  | C34B-H34D∙∙∙O6 | 0.96 | 2.46 | 3.110(16) | 125 | - |

| **Table S3: Selected bond distances (Å) and angles (°) for compounds 1-2** | |
| --- | --- |
| **1** | Zn01-O1, 1.951(2); Zn01-O4, 1.9875(19); Zn01-O7, 2.003(3); Zn01-O6, 2.008(3).  <O1-Zn01-O4 97.79(8); <O1-Zn01-O7 106.87(12); <O4-Zn01-O7 112.37(13); <O1-Zn01-O6 105.71(14); <O4-Zn01-O6 106.72(13); <O7-Zn01-O6 124.00(16). |
| **2** | Cd01-O9, 2.2245(16); Cd01-O1, 2.2702(16); Cd01-O11, 2.3332(16); Cd02-O10, 2.2376(16); Cd02-O2, 2.2674(18); Cd02-O12, 2.297(2); Cd02-O4, 2.3108(17); Cd02-O3 2.4221(17); Cd02-O11, 2.4430(17).  <O9-Cd01-O1 91.21(6); <O9’-Cd01-O1 88.79(6); <O9-Cd01-O11 86.89(6); <O9-Cd01-O11’ 93.12(6); <O1-Cd01-O11’ 88.27(6); <O1-Cd01-O11 91.73(6); <O10-Cd02-O2 137.56(6); <O10-Cd02-O12 86.31(7); <O2-Cd02-O12 90.21(8); <O10-Cd02-O4 139.49(6); <O2-Cd02-O4 82.22(6); <O12-Cd02-O4 104.14(8); <O10-Cd02-O3 86.62(6); <O2-Cd02-O3 135.67(6); <O12-Cd02-O3 89.22(8); <O4-Cd02-O3 55.14(6); <O10-Cd02-O11 85.29(6); <O2-Cd02-O11 89.89(7); <O12-Cd02-O11 167.64(7); <O4-Cd02-O11 88.12(6); <O3-Cd02-O11 99.30(6).  Symmetry code ‘= -x, 1-y, -z |

**References**

Bauer, C. A., Timofeeva, T. V., Settersten, T. B., Patterson, B. D., Liu, V. H., Simmons, B. A., and Allendorf, M. D. (2007). Influence of Connectivity and Porosity on Ligand-Based Luminescence in Zinc Metal−Organic Frameworks. *J. Am. Chem. Soc.* 129, 7136-7144.

Bruker, *APEX2*. Bruker AXS Inc., Madison, Wisconsin, USA, (2012).

Ding, C. X., Li, X., Ding, Y. B., Li, X., Weng, S., and Xie Y. S. (2012). Novel Bis(4,4′-dipyridylamine) Ligand with a Flexible Butadiyne Linker: Syntheses, Structures, and Photoluminescence of d^10^ Metal Coordination Polymers. *Cryst. Growth Des*. 12, 3465-3473.

Farrugia, L. J. (2012). WinGX and ORTEP for Windows: an update**.** *J. Appl. Crystallogr*. 45, 849–854.

Sheldrick, G. M. (1996). SADABS. Program for Empirical Absorption Correction.

Sheldrick, G. M. (2015). Crystal structure refinement with *SHELXL*. *Acta Crystallogr*. C71, 3–8.

Wang, R., Han, L., Jiang, F. L., Zhou, Y. F., Yuan, D. Q., and Hong, M. C. (2005). Three Novel Cadmium(II) Complexes from Different Conformational 1,1‘-Biphenyl-3,3‘-dicarboxylate. *Cryst. Growth Des*. 5, 129-135.

Xu, B., Xie, J., Hu, H.-M., Yang, X.-L., Dong, F.-X., Yang, M.-L. and Xue, G.-L. (2014). Synthesis, Crystal Structure, and Luminescence of Zn/Cd Coordination Polymers with a New Fuctionalized Terpyridyl Carboxylate Ligand. *Cryst. Growth Des.* 14, 1629−1641.
